# Hierarchical processing underpins competition in tactile perceptual bistability

**DOI:** 10.1007/s10827-023-00852-0

**Published:** 2023-05-19

**Authors:** Farzaneh Darki, Andrea Ferrario, James Rankin

**Affiliations:** 1 Department of Mathematics, College of Engineering, Mathematics and Physical Sciences, University of Exeter, Exeter, UK; 2 Biorobotics Laboratory, École Polytechnique Fédérale de Lausanne, Lausanne, Switzerland

**Keywords:** Perceptual bistability, Vibrotactile stimuli, Levelt’s propositions, Competition model, Periodic forcing, Bifurcation analysis

## Abstract

Ambiguous sensory information can lead to spontaneous alternations between perceptual states, recently shown to extend to tactile perception. The authors recently proposed a simplified form of tactile rivalry which evokes two competing percepts for a fixed difference in input amplitudes across antiphase, pulsatile stimulation of the left and right fingers. This study addresses the need for a tactile rivalry model that captures the dynamics of perceptual alternations and that incorporates the structure of the somatosensory system. The model features hierarchical processing with two stages. The first and the second stages of model could be located at the secondary somatosensory cortex (area S2), or in higher areas driven by S2. The model captures dynamical features specific to the tactile rivalry percepts and produces general characteristics of perceptual rivalry: input strength dependence of dominance times (Levelt’s proposition II), short-tailed skewness of dominance time distributions and the ratio of distribution moments. The presented modelling work leads to experimentally testable predictions. The same hierarchical model could generalise to account for percept formation, competition and alternations for bistable stimuli that involve pulsatile inputs from the visual and auditory domains.

## Introduction

**Fig. 1:**
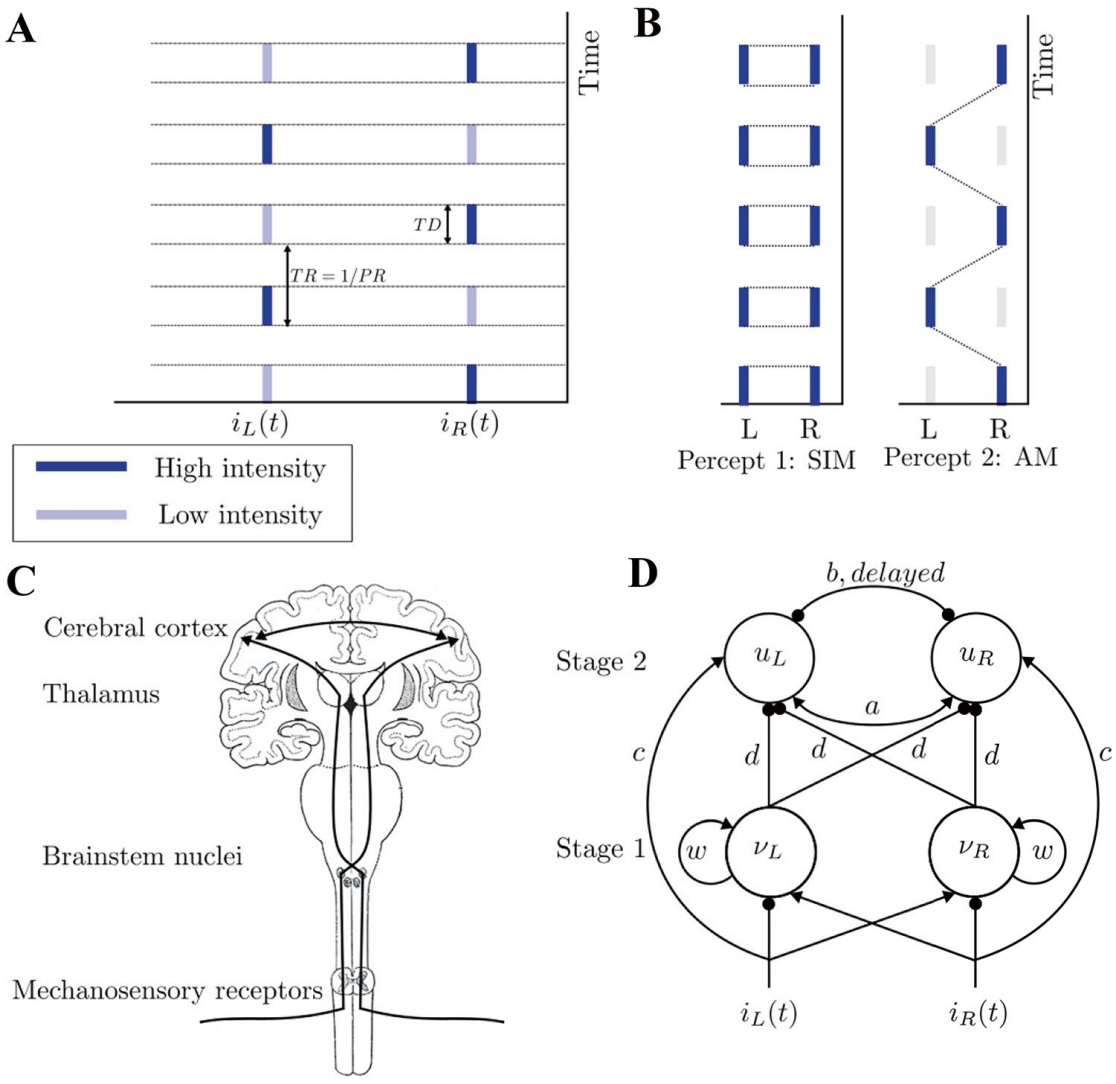
**A Vibrotactile stimuli.** Vibrotactile stimuli consist of antiphase sequences of high (dark blue) and low-intensity (light blue) 400 ms duration, 200 Hz pulses delivered to the right and left index finger each followed by a 400 ms gap, i.e. the pulse duration $TD=0.4$ s and pulse repetition time $TR=0.8$ s. **B Percept types.** During a trial, the participant’s perceptual interpretation of the stimuli changes. When the patterns are played with equal intensity, they are unambiguously perceived as one simultaneous vibration (SIM). With a fixed intensity difference $(\Delta I>0\,dB)$ between the high- and low-intensity tactile pulses, perception switches back and forth between two percepts: SIM (perceived as a fixed intensity on each hand, even though the intensity is changing) and AM (perceived as pulses of vibrations jumping from one hand to the other hand). **C Structure of somatosensory pathway.** Afferent fibres cross over and project to thalamic nuclei on opposite side, then project to cerebral cortex. **D Schematic of the model of tactile rivalry.** Inhibitory connections are shown with filled circles, and excitatory connections with black arrows; see text for definitions of neural populations (units) and their parameters

Perceptual rivalry occurs when sensory information is ambiguous and the brain cannot commit to a single percept; instead, it switches between mutually exclusive interpretations every few seconds Sterzer et al. (2009). Examples of perceptual rivalry span across different sensory modalities including vision (Ramachandran & Anstis, 1985; Blake, 1989; Hupé & Rubin, 2003; Meso et al., 2016), audition Pressnitzer and Hupé (2006) and olfaction Zhou and Chen (2009). In the tactile domain, perceptual rivalry was introduced with a tactile illusion based on the visual apparent-motion quartet (Carter et al., 2008; Conrad et al., 2012; Liaci et al., 2016; Haladjian et al., 2020). Recent experiments with vibrotactile stimuli have shown that several of the general characteristics of perceptual rivalry extend to tactile domain Darki and Rankin (2021). Vibrotactile stimuli consisted of antiphase sequences of high and low intensity high frequency pulses delivered to the right and left index fingers (Fig. 1A). Participants perceived the stimulus (Fig. 1B) as either one simultaneous pattern of vibration on both hands (SIM), or patterns of vibration that jumped from one hand to the other hand, giving a sensation of apparent movement (AM) Ramachandran and Anstis (1985), and for long presentations of the stimulus ($>30\,s$), perception switched back and forth between these two perceptual interpretations (percepts).

There are numerous stimulus examples for perceptual ambiguity across different sensory modalities and across different paradigms within the same sensory modality. In spite of this diversity, general characteristics of this phenomenon appear to be quasiuniversal. Firstly, Levelt’s propositions have been widely used to describe perceptual rivalry in the visual (Moreno-Bote et al., 2010; Brascamp et al., 2015b), auditory Rankin et al. (2015) and recently in tactile domains Darki and Rankin (2021). For example, the generalization of Levelt’s proposition II states that increasing the difference between percept strengths increases the mean perceptual dominance of the stronger percept Levelt (1965). Secondly, despite mean dominance times varying widely in multistable experiments, across different observers and stimulus contrasts (Zhou et al., 2004; Brascamp et al., 2005), the statistical distribution of perceptual phases maintains a constant shape, resembling a log-normal or gamma distribution with consistent values for the coefficient of variation *cv* and skewness ratio ($\gamma _1/cv$; where $\gamma _1$ is the skewness) (Cao et al., 2016; Denham et al., 2018; Darki & Rankin, 2021). Thirdly, the dominance durations of successive percepts are correlated positively for perceptual phases that were one phase apart (between different percepts) (van Ee, 2009; Barniv & Nelken, 2015; Cao et al., 2021; Darki & Rankin, 2021).

These similar properties in multistable phenomena suggest that the underlying mechanisms may be general and is likely to be resolved at a higher level of cognition that is not specific to individual sensory modalities Pressnitzer and Hupé (2006). However, more recent studies suggest that perceptual switching arises from a distributed system of similar but independent processes (Denham et al., 2018; Higgins et al., 2021). There are also considerable debates on whether conscious bistable perception arises within the sensory regions of the brain or within higher-level regions (Snyder et al., 2015; Melloni et al., 2023). On one side of this debate are higher-order theories like global neuronal workspace theory which claim that conscious state critically depends on global broadcasting of information across an interconnected network of prefrontal-parietal areas and many distant high-level sensory cortical areas. On the other side of this debate are sensory theories such as integrated information theory, which claim that the true neural correlates of perceptual awareness is located in more posterior, sensory regions of the brain Hatamimajoumerd et al. (2022). According to these theories, activations in the frontal lobe are not associated with the conscious perception of the stimulus but are instead associated with the post-perceptual processes associated with reporting that stimulus (e.g., attention, decision-making, motor outputs, etc.) Cohen et al. (2020). To ensure that the resulting differential neural activity is exclusively associated with perceptual awareness and not with post-perceptual processing no-report paradigms are developed in which observers are aware or unaware of a stimulus, but they do not make any explicit post-perceptual judgments about that stimulus Brascamp et al. (2015a).

Computational models of perceptual alternations have helped significantly with our understanding of sensory processing across visual and auditory domains. These models focus on the neural processing of sensory information, the dependence of average switching times on stimulus parameters and the statistical distribution of times between switches (Rankin et al., 2015; Brascamp et al., 2005; Laing & Chow, 2002; Shpiro et al., 2007; Wilson, 2003; Moreno-Bote et al., 2007; Vattikuti et al., 2016; Li et al., 2017). General models of rivalry usually incorporate a slow process together with reciprocal inhibition to produce perceptual alternations. Perceptual bistability results from competition between units representing neural populations associated with different percepts. For example, in the visual domain, models presented for binocular rivalry consider distinct neural populations encoding features (like orientation of gratings) for the left and right eyes (Wilson, 2003; Li et al., 2017; Cao et al., 2021). In the auditory domain, models for auditory streaming represent competition between neural populations driven by tonotopic responses from primary auditory cortex (Rankin et al., 2015; Ferrario & Rankin, 2021). A more recent model of auditory streaming includes competitive dynamics through multiple hierarchical levels of processing Little et al. (2020). A new concept suggests that perceptual bistability is not solely derived from one source, but instead, it is influenced by numerous sources of adaptation, inhibition, and noise distributed throughout the brain Higgins et al. (2020, 2023). Bifurcation analysis has been used with these models to compute different dynamical regimes and boundaries between them for multiple parameters with fixed Shpiro et al. (2007) and periodic stimuli Darki and Rankin (2020).

Here, we address the need for a tactile rivalry model (to the best of our knowledge, one is yet to be proposed) that accounts for well-established results on the duration of dominance intervals and incorporates the structure of the somatosensory system based on physiological evidence. In the tactile domain, competition arises between neural populations encoding responses to pulsatile stimulation, with patterns of high and low intensity, at distinct points on the skin. It remains an open modelling challenge to address how sequences of pulsatile inputs are integrated and encoded as percepts, and how neural competition resolves ambiguity to select and switch between percepts. Existing models with pulsatile inputs, developed for the visual and auditory domain (Wilson, 2003; Rankin et al., 2015; Li et al., 2017), did not address the competition mechanisms that link between inputs separated in time and across a feature (e.g. between left and right eyes in binocular rivalry or across a tone frequency in auditory streaming). A recent study (upon which we build) went some way to addressing the question of percept formation in auditory streaming, but did not address switching between percepts, the dependence of average switching times on stimulus parameters (like Levelt’s propositions) and the statistical distribution of times between switches Ferrario and Rankin (2021). Interaction of adaptation and delayed inhibition allows for some neuronal memory that links each pulse to the next one from two different sources on the skin to form a coherent representation of the pulsatile stimuli.

In this study, we develop a mathematical model of tactile rivalry that focuses on accurately reproducing the dynamics of the perceptual alternations. The model is neuromechanistic, i.e. based on computational principles widely accepted as underpinning cortical processing. This formulation is directly motivated from physiological studies of tactile perception (Noachtar et al., 1997; Maldjian et al., 1999; Nihashi et al., 2005), a model of bistable dynamics Levenstein et al. (2019), and a model of auditory percept formation for sequences of pulsatile inputs (i.e. tones) Ferrario and Rankin (2021). The novel combination of these mechanisms addresses shortcomings of earlier models by simultaneously addressing the formation of percepts via mechanisms that link individual stimulus elements across time, switches between the encoded percepts and statistical characteristics of dominance duration distributions. The model of tactile rivalry presented here consists of two processing stages; first stage for producing perceptual alternations; and a second stage for encoding the percept types (SIM and AM). The powerful combination of bifurcation analysis with periodic forcing along with optimisation tools have been used to tune certain features of the model.

The model presented here produces experimentally-observed characteristics of tactile rivalry, including the specific temporal structure of the percepts and switching between them. We offer predictions in terms of how left-right tactile intensity differences are encoded and the putative location of percept encoding in somatosensory cortex. The model provides a framework to predict parameter dependence of dominance duration for future behavioural work. The same framework and hierarchical structure can generalise to the study of other perceptually bistable stimuli involving sequences of pulsatile inputs as investigated elsewhere in auditory Rankin et al. (2015) and visual paradigms (Wilson, 2003; Li et al., 2017).

## Methods

### Percepts and model inputs

In tactile rivalry experiments Darki and Rankin (2021), stimuli consisted of sequences of high (H) and low (L) intensity vibratory pulses, each followed by a silent interval (antiphase sequences of $H-L-H-L$ for the right hand and $L-H-L-H$ for the left hand, “−” indicates the silent gap) (Fig. 1A). The intensity of the *L* stimulus is $\Delta I$ below the intensity of the H stimulus on a logarithmic scale (*dB*). During experimental trials of tactile rivalry, these stimuli are perceived as either one simultaneous pattern of vibration on both hands (SIM), or patterns of vibration that jumped from one hand to the other hand, giving a sensation of apparent movement (AM) (Fig. 1B), and perception switches back and forth between these two interpretations.

Model input functions, $i_{R}(t)$ and $i_{L}(t)$ as defined by Eqs. (1) and (2), are antiphase periodic square waves Ferrario and Rankin (2021) corresponding to the stimuli delivered to right and left hands, respectively.

1 $$\begin{aligned} \begin{aligned}&i_{R}(t) = \sum _{k=0}^{\infty } \chi _{I_{high}^{k}}\!\!(t) + (1-\Delta I) \sum _{k=0}^{ \infty } \chi _{I_{low}^{k}}\!\!(t), \\&I_{high}^{k}=[2kTR, 2kTR+TD], \\&I_{low}^{k}=[(2k+1)TR, (2k+1)TR+TD]. \end{aligned} \end{aligned}$$

2 $$\begin{aligned} \begin{aligned}&i_{L}(t) = (1-\Delta I) \sum _{k=0}^{\infty } \chi _{I_{low}^{k}}\!\!(t) + \sum _{k=0}^{ \infty } \chi _{I_{high}^{k}}\!\!(t), \\&I_{low}^{k}=[2kTR, 2kTR+TD], \\&I_{high}^{k}=[(2k+1)TR, (2k+1)TR+TD]. \end{aligned} \end{aligned}$$

Where $\Delta I$ represents the intensity difference between high and low amplitude vibratory pulses. $\chi _{I}$ is the standard indicator function over the set of intervals *I*, defined as $\chi _{I}(t)=1$ for *t* in *I* and 0 otherwise. The intervals when high and low intensity vibrations are on are respectively given by $I_{high}^{k}$ and $I_{low}^{k}$. The parameter *TD* represents the duration of high or low intensity pulses, and *TR* is the time between pulse onsets ($PR=1/TR$ is the presentation rate); see a schematic plot of the stimulus in Fig. 1A and refer forward to a plot of one period of the stimulus in Fig. 8A.

In order to have a smooth square waveform rather than an ideal discontinuous square waveform, we used a steep sigmoid,

3 $$\begin{aligned} {R}(i) = \frac{1}{{1+e^{ -k(i)}}} \end{aligned}$$

with k=20 which defines the slope. So instead of the ${i_R}$ and ${i_L}$, we substitute $R({i_R})$ and $R({i_L})$ in the model’s inputs.

### Processing stages

**Fig. 2:**
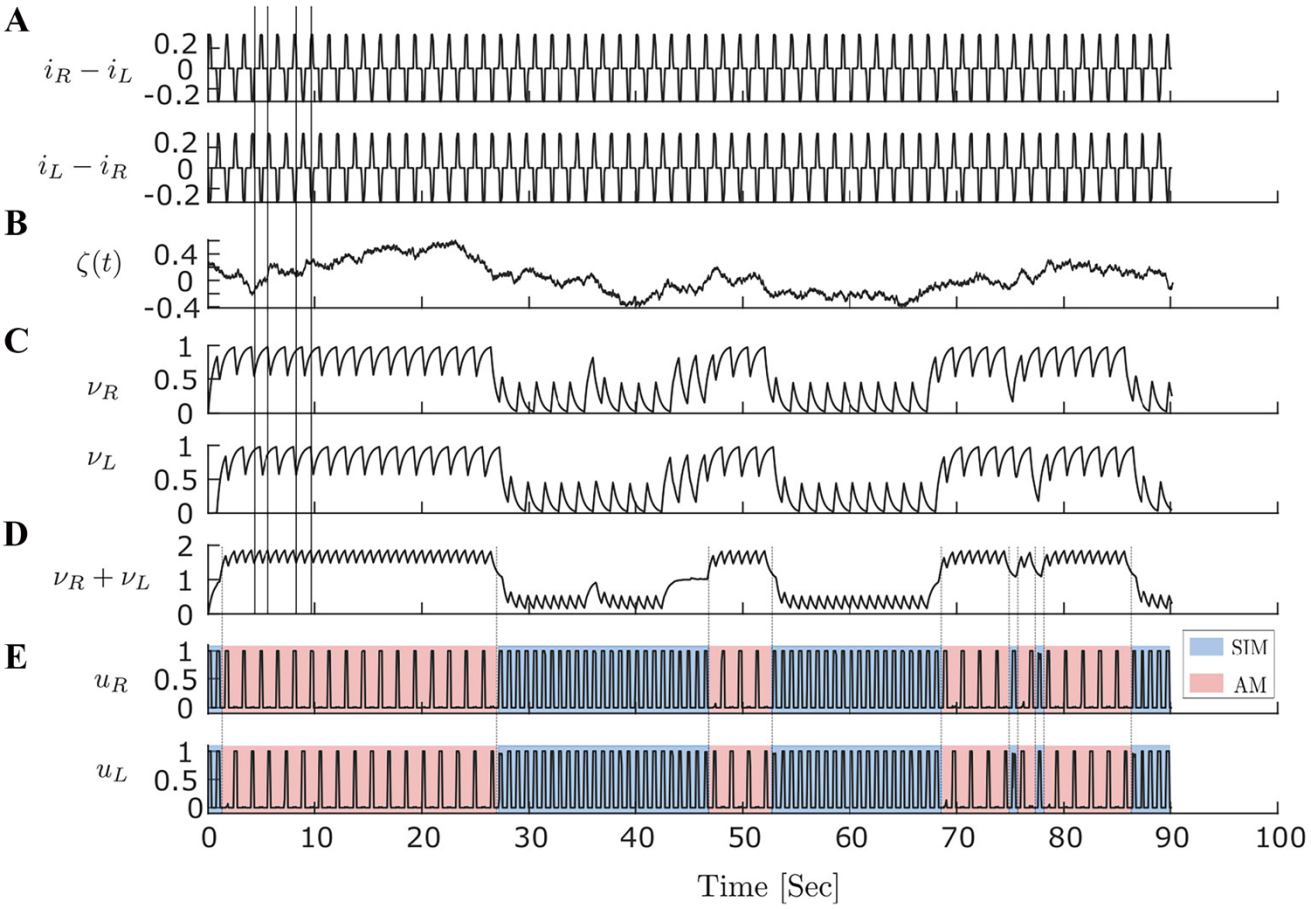
**Time histories of tactile rivalry model.** Population firing time responses at $\Delta I=2\,dB$ for 90 s simulation. **A** Net inputs to the left (top panel) and right (bottom units of the first stage). **B** Noise with parameters $\sigma =0.3$ and $\tau _n=0.05$ which is added to the first stage inputs. **C** Firing activities of the first stage units to the inputs in panel A and noise in panel B. Vertical lines are plotted to show that unit activities in the first stage are antiphase due to their antiphase inputs. The activity decays when the input is negative and increases otherwise. **D** Sum of firing activities of the first stage units, which will be the inhibitory input to both units of the second stage. As mean firing activities in the first stage are antiphase, there is low amplitude oscillation when they are summed up. **E** Firing activities of the second stage units to the inputs in panel D. Perceptual alternation between SIM and AM percepts in the seconds stage are seen as transition occurs between DOWN and UP states in panel D

Here we want to introduce a computational model of tactile rivalry to capture the characteristics of this phenomenon as observed in the experiments. To this end, we consider how inputs from the left and right hands project to somatosensory cortex and how features like amplitude, frequency and timing are encoded there (Fig. 1C). The model presented here consists of two processing stages (Fig. 1D); a first stage with units $\nu _L$ and $\nu _R$, and a second stage with units $u_L$ and $u_R$. The two stages receive the inputs defined above in parallel. Contra-lateral excitation and ipsi-lateral inhibition are shown in Fig. 1D, as inputs $i_R$ and $i_L$ have an excitatory effect on the opposite side (excitation of units $\nu _L$ and $\nu _R$, respectively), and inhibitory effects on the same sides (inhibit units $\nu _R$ and $\nu _L$, respectively). Thus, units $\nu _R$ and $\nu _L$ in the first stage effectively receive $i_L-i_R$, and $i_R-i_L$, respectively, which are antiphase pulses with amplitude equal to $\Delta I$ (Fig. 2A).

The units $\nu _R$ and $\nu _L$ in the first stage are firing rate models with recurrent excitation (with strength $\omega$) and slower recurrent adaptation (with strength *g*). These units produce alternations between an UP (ON) and DOWN (OFF) state. This model repurposed as a component in the present study has previously been used to investigate the state of hippocampal and neocortical populations during NREM sleep (UP/DOWN states observed as spontaneous transitions during sleep) Levenstein et al. (2019). The analysis in their study provides a useful reference to tune model parameters. The second stage in Fig. 1D shows units $u_R$ and $u_L$ that receive direct ipsi-lateral excitatory inputs from the right and the left side ($i_R$ and $i_L$ weighted with strength *c*, respectively), and also inhibitory ipsi- and contra-lateral connections with strength *d* through the earlier stage (units $\nu _R$ and $\nu _L$). Interhemispheric connections at the second stage are assumed to exist between units $u_R$ and $u_L$ through direct fast excitation with strength *a*, and the delayed, slowly decaying inhibition with strength *b*.

When the first stage units are in the DOWN state, inputs drive the second stage in its default setting where typically SIM is encoded, unless $\Delta I$ is very large. If the first stage units are in the UP state, inputs driving the second stage are less excitatory, leading to AM, unless $\Delta I$ is very small. In other words, the first stage computes amplitude differences between the left and right inputs and amplifies these differences via global inhibition of the second stage (strength *d*); however, this effect is transient due to adaptation (a slow negative recurrent feedback) on each unit $\nu _L$ and $\nu _R$, which leads to alternations. As described in more detail below, alternations in the first stage lead to switches in the percept encoded by the second stage due to changes in the strength of global inhibition via *d*. The dynamics of the units model in the first and second stages are described in more details in Appendix.

### Full tactile rivalry model with noise

To form the model of tactile rivalry, the model encoding alternations at the first stage is incorporated with the model encoding percepts at the second stage. Units $\nu _R$ and $\nu _L$ in the first stage make inter- and intra-hemispheric inhibitory connections with the units $u_R$ and $u_L$ in the second stage (Fig. 1D).

4 $$\begin{aligned} \left\{ \begin{array}{l} \begin{aligned} {\tau _\nu }{\dot{\nu }_R}=-{\nu _R}+N(w\,\nu _R&{}-g\,\alpha _R\\ &{}+f(\Delta I)\,\frac{i_{L}- i_{R}}{\Delta I} +\zeta (t)),\\ {\tau _\nu }{\dot{\nu }_L}=-{\nu _L}+N(w\,\nu _L&{}-g\,\alpha _L\\ &{}+f(\Delta I)\,\frac{i_{R}- i_{L}}{\Delta I}+\zeta (t)),\\ \tau {\dot{u}}_{R}=-{u_{R}}+H(a\,u_{L}&{}-b\,x_{L}\\ &{}-d\,(\nu _R+\nu _L)+c\,i_{R}-\theta ),\\ \tau {\dot{u}}_{L}=-{u_{L}} +H(a\,u_{R}&{}-b\,x_{R}\\ &{}-d\,(\nu _R+\nu _L)+c\,i_{L}-\theta ). \end{aligned} \end{array} \right. \end{aligned}$$

The dynamics of units in the first stage are described in terms of the mean firing rates $\nu _R$ and $\nu _L$ with time scale $\tau _\nu$, and activity-driven adaptation $\alpha _R$ and $\alpha _L$ with time scale $\tau _\alpha$ (Fig. 1D). Where *w* is the strength of recurrent excitation, *g* is the strength of adaptation, $(i_{L}- i_{R})$ and $(i_{R}- i_{L})$ are the stimulus differences from the right and left, and $\zeta (t)$ is noisy fluctuations. The nonlinear function $f(\Delta I)$ will be determined later with data-driven optimisation. *N*(*x*) is assumed to be sigmoidal activation function as follows;

5 $$\begin{aligned} {N}(x) = \frac{1}{{1+e^{ -(x - {x_0})}}}. \end{aligned}$$

The dynamics of units in the second stage are described in terms of the mean firing rates $u_{R}$ and $u_{L}$ of two neural populations which encode sequences of vibratory input pulses with timescale $\tau$. The synaptic variables $x_{R}$ and $x_{L}$ describe the time-evolution of inhibitory dynamics through indirect synapses that can generate delays approximately equal to $\delta$ Rubin and Terman (2000) (see Appendix). The Heaviside gain function *H*(*x*) is equal to 1 for $x>=0$, and 0 for $x<0$. Mutual coupling through direct fast excitation has strength *a*. The delayed, slowly decaying inhibition has timescale $\tau _i$, strength *b*. The strength of inhibitory connections between the first and second stage is *d*. The model is driven by excitatory inputs $i_R(t)$ and $i_L(t)$ with strength *c*.

We extended the previous model proposed by Ferrario and Rankin Ferrario and Rankin (2021) by transforming the delayed inhibition into a system of ordinary differential equations using the approach described in Rubin and Terman (2000). This in turn allowed us to track periodic orbits modulated by forcing under parameter variation (as described in Darki and Rankin (2020) and using numerical continuation with Auto07p). Note that this approach only works for small to moderate delays.

Noise $\zeta (t)$ was implemented using Ornstein-Uhlenbeck model described by

6 $$\begin{aligned} {d\zeta } = -\tau _n \zeta dt + \sigma \sqrt{2\tau _n dt }W_{t} , \end{aligned}$$

where $W_{t}$ is a Wiener process with time scale $\tau _n$ and standard deviation $\sigma$.

### Bifurcation and statistical analysis

The Heaviside function *H*(*x*) that appears in the right-hand side of the full tactile model is a discontinuous function in its first derivative. Numerical continuation routines require smooth systems of equations. In order to solve this problem we have used a steep sigmoid function to smooth out the transition at zero.

7 $$\begin{aligned} {S}(x) = \frac{1}{{1+e^{ -k(x)}}} \end{aligned}$$

with k=20 which defines the slope. So instead of the *H*(*x*) , we substitute *S*(*x*) in the right-hand side of the full tactile model.

Bifurcation analysis of the model in the absence of noise was carried out with Auto07p [Source code for the model is available in the GitHub repository farzaneh-darki/Darki2022-hierarchical: https://github.com/farzaneh-darki/Darki2022-hierarchical]. For the statistical analysis of dominance duration distributions, the same model was implemented in MATLAB for simulations with noise. Numerical integration of the resulting stochastic differential equation was carried out using a standard Euler-Muruyama scheme with time step 0.01 ms which is much smaller than the fastest timescale ($\tau =0.001$ s=1 ms). All the model parameters and their corresponding values are provided in Table 1.

**Table 1:** Parameters of the model with their corresponding values used in the simulations. The main value of the model parameters are provided here. If a parameter changes from its main value, the new value is determined in the relevant figure

Parameters	Values	Parameters	Values
*w*	6	*a*	3.4
*g*	1.5	*b*	2.8
${\tau _\nu }$	0.9	*c*	5.5
${\tau _\alpha }$	4.5	$\delta$	0.005
${x_0}$	5	$\tau _i$	0.25
$k_A$	15	$\tau$	0.001
${\nu _0}$	0.5	$\theta$	0.5
$\sigma$	1	$\alpha _{x}$	50
$\tau _{n}$	0.05	$\beta _{x}$	8
*d*	2.6	$\theta _{s}$	0.22

### Simplified tactile rivalry model

**Fig. 3:**
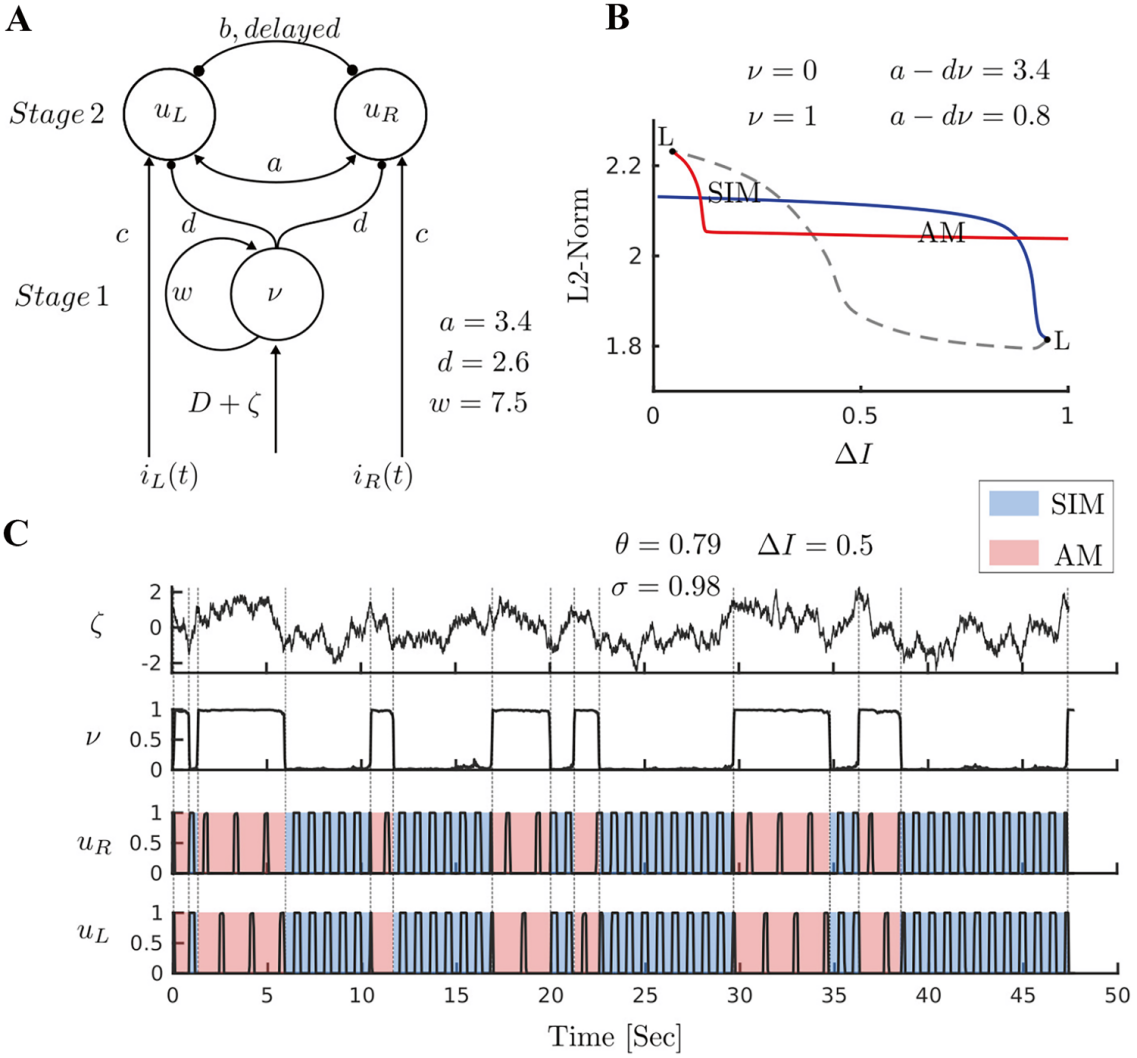
**Mechanism of perceptual alternations.** **A Simplified model of tactile rivalry.** The adapting recurrent model with firing rate $\nu$ (analysed in isolation in Fig. 6) makes inhibitory connections with strength *d* to the units encoding the percepts $u_R$ and $u_L$ (analysed in isolation in Fig. 8). **B Bifurcation analysis with respect to intensity difference** $\Delta I$. There is a region of bistability between two fold of limit cycle bifurcation points (L). Branches of periodic orbits associated with SIM and AM percepts coexist at this interval. **C Time histories of model responses.** Population activities are simulated for 50 s at $\Delta I=0.5$ ($D=1.25$). Noise realization (top panel), UP/DOWN alternations of the first stage unit driven by noise (second panel, $g=0$), firing activities of the second stage unit (two bottom panels). Perceptual switching times are shown between SIM (blue) and AM (red) with dashed lines

The full tactile rivalry model presented above was inspired by the somatosensory pathways. However, as the inputs $(i_{L}- i_{R})$ and $(i_{R}- i_{L})$ are antiphase, and due to symmetry in the first stage, the model can be simplified. The simplification described below facilitated a detailed analysis of the appropriate combination of mechanisms and parameters that generate dynamics consistent with perceptual interpretations of the stimulus and switching. To this end, units $\nu _R$ and $\nu _L$ can be replaced by one adapting recurrent model with variables $\nu$ and $\alpha$ and input $D=f(\Delta I)$ (Fig. 3A). Where $\Delta I$ is a positive constant, and *f* is a nonlinearity to be determined by data-driven optimisation. So the simplified tactile rivalry model is described by

8 $$\begin{aligned} \left\{ \begin{array}{l} {\begin{matrix} &{}{\tau _\nu }{\dot{\nu }} = - {\nu } + {N}\left( { w\,\nu - g\,\alpha + D +\zeta (t)} \right) ,\\ &{}\tau {\dot{u}}_{R} = - {u_{R}} +H(a\,u_{L}-b\,x_{L}+c\,i_{R}-d\,\nu -\theta ),\\ &{}\tau {\dot{u}}_{L} = - {u_{L}} +H(a\,u_{R}-b\,x_{R}+c\,i_{L}-d\,\nu -\theta ).\\ \end{matrix}} \end{array} \right. \end{aligned}$$

### Bistability in the simplified model

Analysis of the model’s component stages provided a means to tune parameters of the more tractable simplified model, which could then be used in the full model to capture the desired dynamics, as will be discussed in the results section. Full details of the analysis of each stage is given in Appendix. The general aim was to tune parameters so as to produce a region of bistability between states representing SIM and AM in the noise-free simplified model. The introduction of noise can then drive alternations between these states. Figure 3B shows a bifurcation diagram with the desired coexistence of SIM and AM solution branches over a significant range of $\Delta I$. Figure 3C shows a time history of the noise-driven simplified model, where alternations in the first stage (as $\nu$ transition occurs from zero to one or vice versa) drive switches between a synchronised SIM state (blue) and antiphase oscillations (AM). Population activities are simulated for 50 s at $\Delta I=0.5$. The top panel shows the noise realization with parameters $\sigma =0.79$ and $\tau _n=0.98$.

For the interested reader, a brief summary of the bifurcation analysis of the component stages leading to Fig. 3B is discussed here, with full details given in Appendix. The adapting recurrent model in the first stage makes inhibitory connections with strength *d* to the model encoding the percepts in the second stage (Fig. 3A). As seen in (Fig. 6B & D) unit $\nu$ has a region of bistability where $\nu$ can be either zero or one (when input *D* lies between two fold bifurcation points). As this unit inhibits $u_L$ and $u_R$ with strength *d*, the convergence to 0 or 1 of this unit will modify the excitatory net inputs to units $u_L$ and $u_R$ and thus shift the branch of periodic orbits in Fig. 3B to the right and to the left (when $\nu =1$, second stage receives less excitation, effectively $a_{\text {eff}}=a-d\nu$; see Fig. 8E). This results in bistability between SIM and AM dynamical states shown in the bifurcation diagram of the whole model (Fig. 3B).

## Results

Here we go through a qualitative description of the dynamics produced by the full tactile rivalry model presented in Eq. (4) and explain how this qualitatively matches perceptual interpretations and alternations observed in tactile rivalry experiment. We further analyse the dependence of mean dominance durations and their variability (as characterised by a skewed distribution) on the stimulus parameter $\Delta I$.

### Time history simulations of full tactile rivalry model

We first discuss the output from individual simulations of the model and illustrate how model’s firing rate variables can encode the competing percepts and perceptual alternations. A region of bistability, identified by a detailed bifurcation analysis of the model, was described in methods section above (Fig. 3). For the interested reader, a detailed analysis of the model and it’s component stages as given in Appendix shows how the tactile rivalry model was designed to encode percepts and generates perceptual alternations.

A 90 s time simulation for the full tactile rivalry model is shown in Fig. 2. The units in the first stage are excited by the contra-lateral stimulus and inhibited by the ipsi-lateral stimulus. Thus, the net inputs to the left and right units of the first stage will be the contra-lateral stimuli minus ipsi-lateral stimuli. These inputs are antiphase pulses with amplitude proportional to $\Delta I$ as shown in Fig. 2A. These inputs weighted by $f(\Delta I)$ and delivered to the units in the first stage. Noise is added to these inputs with amplitude $\sigma =0.3$ and timescale $\tau _n=0.05$ (Fig. 2B). Firing activities of these units in the first stage in response to the stimuli and noise are shown in Fig. 2C. In the absence of noise, these adapting recurrent units could oscillate between the UP and DOWN states regularly. However, these oscillations are now driven by both adaptation and noise process, and irregular oscillations are observed in Fig. 2C between UP and DOWN states. Figure 2D shows the sum of firing activities of the first stage, which is delivered as an inhibitory input to both units of the second stage. Firing activities of the second stage units to these inputs are shown in Fig. 2E. Units of the second stage encode the SIM percept (both units fully respond to the high and low intensity pulses in the inputs) when there is low level of inhibition from the first stage (Fig. 2D). When the level of inhibition crosses a certain threshold (marked by vertical dashed lines in Fig. 2D & E), the units in the second stage encode AM percept (both units only fully respond to the high intensity pulses in the inputs). Perceptual alternation between the SIM and AM percepts are seen as transitions between DOWN and UP states occur in the inhibitory inputs (Fig. 2D & E). A combination of adaptation and noise can decrease the level of bilateral inhibition which leads to a transition from SIM to AM.

### Stimulus parameter dependence

**Fig. 4:**
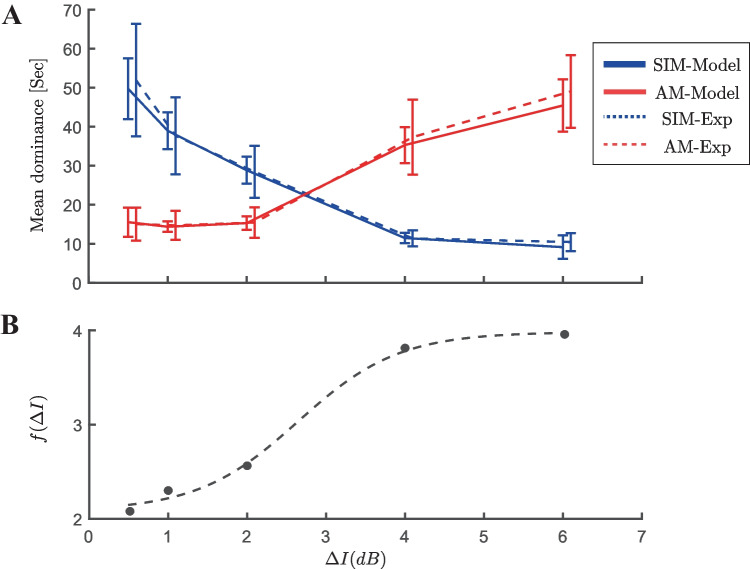
**Levelt’s proposition II.** **A** Experimental data are dashed curves and computational data from the model are solid curves with data points at different values of intensity difference $\Delta I=0.5, 1, 2, 4, 6$ on the x-axis, error bars show standard error of the mean. Mean dominance duration of the SIM percept (blue) decreases as the intensity difference increases, while an opposite effect is observed for the AM percept (red). **B** Nonlinearity in the inputs of the first stage ($D=f(\Delta I)$) are determined using an optimisation algorithm. Dashed black curve is the best fit for an offset and scaled sigmoid nonlinearity. The Experimental data is obtained from Darki and Rankin (2021)

Results from experiments with vibrotactile stimuli demonstrate that Levelt’s proposition II holds in tactile domain Darki and Rankin (2021). Increasing intensity difference, causes the mean dominance of SIM percept to decrease and AM percept to increase. Mean dominance duration for both the perceptual durations from the model and the experiment (Experimental data from Darki and Rankin (2021)) are plotted against intensity difference ($\Delta I$) in Fig. 4. The parameters of the noise ($\sigma$, $\tau _n$), time constant of adaptation ($\tau _a$), and nonlinearity in the inputs of the first stage ($D=f(\Delta I)$) were determined using a genetic algorithm. Our optimisation approach also determined the nonlinearity *f*. The good match with experimental data, obtained by tuning a small number of parameters and the input nonlinearity offers confidence that the model presented here is an effective, parsimonious description of the potential mechanisms driving tactile rivalry.

To find nonlinear function $f(\Delta I)$, we first estimated some points of it at the experimental conditions ($\Delta I=0.5, 1,2,4,6\,dB$) using a genetic algorithm ($f(0.5)=2.16$, $f(1)=2.3$, $f(2)=2.6$, $f(4)=3.75$, $f(6)=3.92$ with $\tau _n =0.05$, $\sigma =0.3$, $\tau _a=5\,s$). Having these points, we showed an offset and scaled sigmoid function like;

9 $$\begin{aligned} f(\Delta I) = f_0+\dfrac{f_1}{1+\exp (-r(\Delta I-\Delta I_0))}, \end{aligned}$$

with parameters: $f_0=2.12$ (offset), $f_1=1.80$ (scale), $r=1.57$ (slope), $\Delta I_0= 2.64$ (equidominance), fits best to these points.

### Variability of perceptual durations

**Fig. 5:**
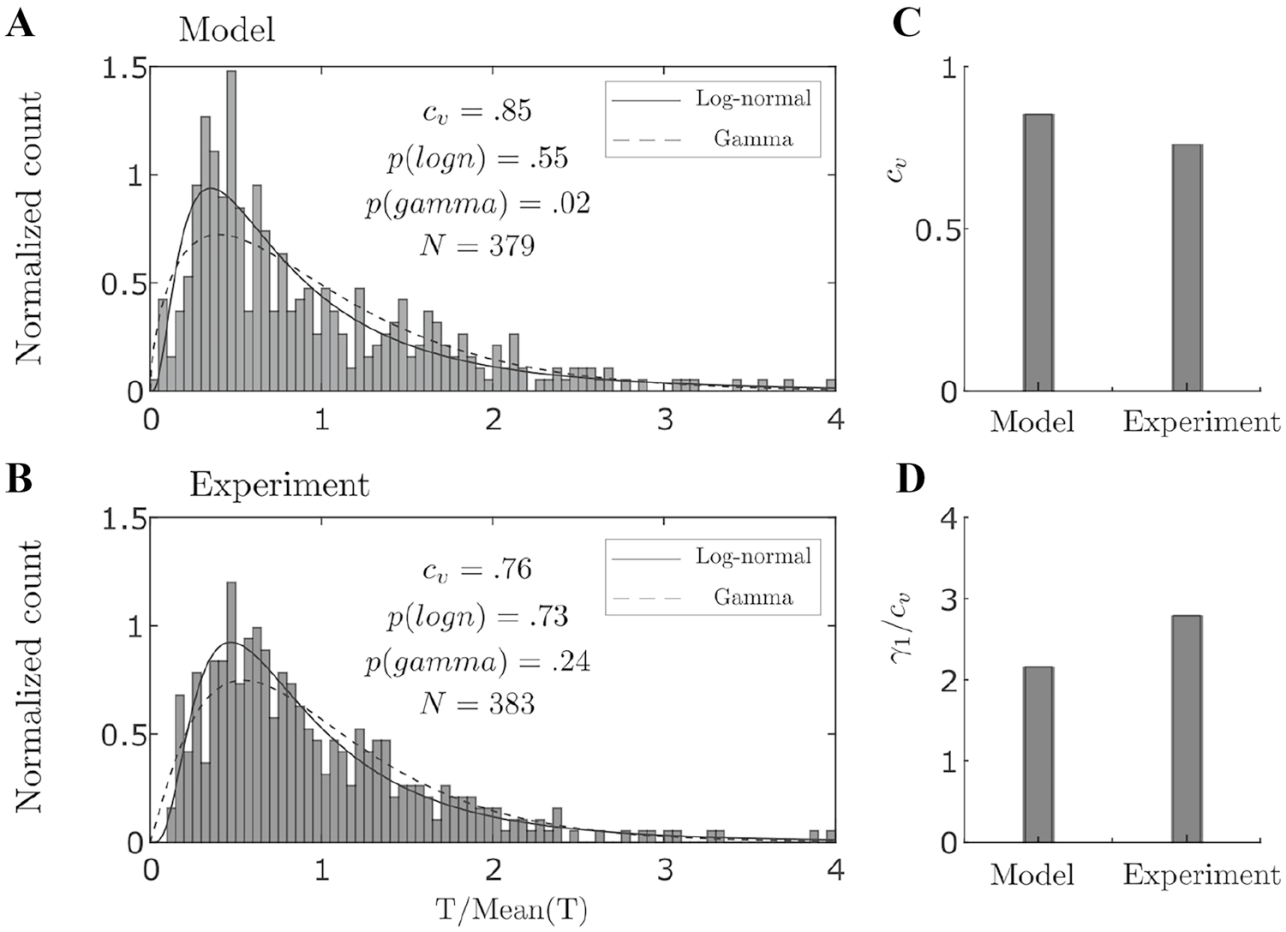
**Statistics of dominance durations.** **A Model.** Histogram of 379 durations from model simulations at $\Delta I=2\,dB$ combined across perceptual type after normalising by the mean. Solid and dashed curves show the estimated log-normal and gamma distribution, respectively. P-values are from one-way KS test. **B Experiment.** Histogram of normalized perceptual durations combined across participants and percept type after normalization by the mean, for experimental conditions close to equidominance ($\Delta I=2\,dB$). **C–D Moment ratios.** (**C**) Coefficient of variation ($c_v$) and (**D**) skewness divided by coefficient of variation ($\gamma _1/c_v$) computed for distributions from the model and the experiment at intensity difference $\Delta I=2\,dB$

**Fig. 6:**
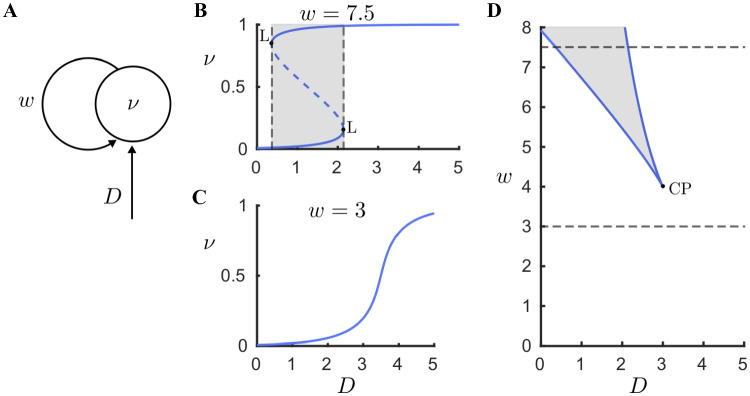
**Bifurcation analysis of the adapting recurrent model (without adaptation** $g= 0$**).** Mechanism of bistability with sufficient levels of recurrent excitation and input (high levels of recurrent excitation and low levels of input). **A Adapting recurrent model.** Population firing rate $\nu$ driven by *D* and recurrent excitation with strength *w*. **B **&** C One parameter bifurcation diagram.** Population rate steady state as a function of input *D*, for a population with low ($w= 3$), and high ($w = 7.5$) level of recurrent excitation. Stable and unstable fixed points are indicated with solid and dashed curves, respectively. **D Two parameter bifurcation diagram.** $w-D$ parameter space, bistable region (grey shaded area) surrounded by two fold bifurcations (L) which merge together and disappear through a cusp bifurcation (CP)

**Fig. 8:**
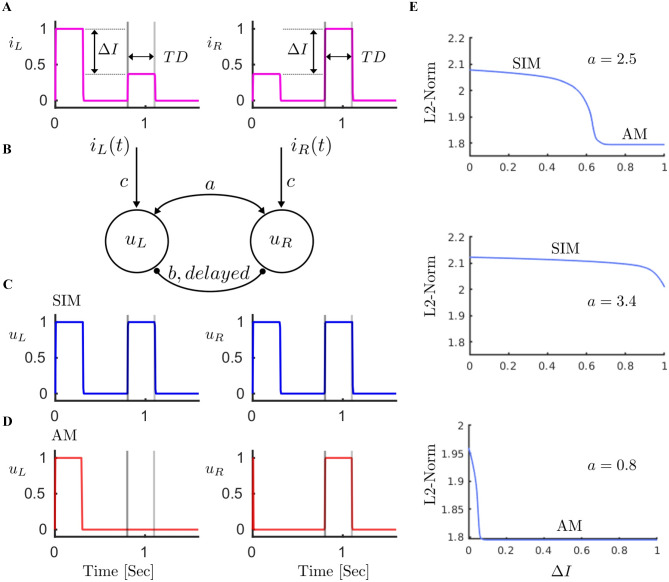
**Mechanism of encoding percepts.** **A Stimuli.** One period (length $2TR=1.6\,s$) of the stimuli to the left and right units consists of one full amplitude pulse (length $TD=0.3\,s$), and one pulse that its amplitude is $\Delta I$ below the intensity of the full amplitude pulse on a logarithmic scale (*dB*). **B Model encoding percepts.** Schematic of the model consisting of mutual fast excitation with strengths *a* and delayed inhibition with strength *b*. Inhibition is delayed of the amount $\delta$. **C SIM percept.** Model can encode percept SIM, when $u_L$ and $u_R$ have full response to both pulses. **D AM percept** is encoded when they only respond to the full amplitude pulse and no response to the low intensity pulse. **E Bifurcation analysis with respect to intensity difference** $\Delta I$. One parameter bifurcation diagram sketched at three different values of *a*. Blue curve shows the branch of periodic orbit, in which there is a sharp transition between periodic respond encodes SIM and AM percepts (top panel). For higher values of excitation (middle panel) this boundary moves to the right, and for lower values of excitation (bottom panel) moves to the left

The distributions of normalized perceptual durations from the model and from the experiment are shown in Fig. 5A & B. These distributions were compared with gamma and log-normal distributions using a one-way Kolmogorov-Smirnov (KS) test. The null hypothesis is that the test data are drawn from the standard comparison distribution and a significant result ($p < .05$) indicates that the test data are not drawn from the comparison distribution. The one-way KS tests shows that the results produced by the tactile rivalry model best fit by a log-normal distribution, but that the gamma distribution can be rejected ($p(gamma) < .05$). For the experimental data, neither distribution could be rejected. However, in similar experiments with auditory bistability (Rankin et al., 2015; Denham et al., 2018) and visual bistability Denham et al. (2018) a log-normal distribution provided a better fit than the gamma distribution. We suspect that increasing the number of participants, or number of trial repetitions, may offer a more conclusive result for tactile rivalry in the future.

To assess how well tactile rivalry model conforms to the moment distribution ratios reported in Darki and Rankin (2021), the statistical characteristics are compared across the model and the experiments. As Fig. 5C & D shows coefficient of variation for the distribution from the model is $c_v=.85$, and the ratio of skewness and coefficient of variation is $\gamma _1/c_v=2.17$, which are comparable to the corresponding values from experiment ($c_v=.76$, $\gamma _1/c_v=2.79$), see discussion section for more details.

## Discussion

Here, a two-stage model of tactile rivalry is introduced that encodes the temporal dynamics and features of both percepts observed in tactile rivalry experiments, and alternations between these percepts. Bifurcation analysis was used to tune model parameters for the first stage to operate within bistable or oscillatory regime. And the second stage model parameters are tuned to operate within a range where direct transitions between SIM and AM are possible. Other model parameters have been estimated through a genetic algorithm with a cost function to minimise the differences between the experimental and computational mean dominance curves with respect to intensity difference. The powerful combination of bifurcation analysis to tune certain features of the model, along with optimisation tools, allowed for the design of a model that captures many features from the experiments of tactile rivalry.

### Physiological basis of tactile rivalry model

The somatosensory cortex consists of several neighbouring, functionally distinct areas whose interconnections are complex and only partially understood. Primary somatosensory cortex (S1) and secondary somatosensory cortex (S2) are two major areas of the somatosensory cortex. Right and left afferent fibres go up through the spinal cord, cross over at the brainstem nuclei, and project to the opposite side of thalamus nuclei, and from there project to area S1, preserving the somatotopic organisation Fitzpatric (2008). The majority of afferent fibres in area S2 come from S1, but it also receives direct inputs from the thalamus Friedman and Murray (1986). Furthermore, the body is bilaterally represented in S2, but with the contralateral side dominant Hoechstetter et al. (2001).

The literature illuminates details of excitatory-inhibitory interactions in area S1. Neurons in area 3b of S1 have been characterized using linear spatial receptive fields with spatially separated excitatory and inhibitory regions Delhaye et al. (2011). In addition to an inhibitory component flanking the excitatory one, receptive fields tend to also comprise an inhibitory component co-localized with the excitatory field but delayed by 20 to 30 ms (Gardner & Costanzo, 1980; DiCarlo & Johnson, 2000). This receptive field structure results in an initial excitatory drive that is followed by an inhibitory one, rendering the neuron less excitable for a period of time Delhaye et al. (2011). It is reasonable to assume that similar excitatory-inhibitory mechanisms, detailed above for S1, are replicated downstream in S2, thus motivating the inclusion of intra-cortical fast excitation and delayed inhibition in the model (a delay of 5 ms coupled with slow activation of inhibition in the second stage of model leads to delayed inhibition comparable to observations from somatosensory cortex). In area 3b of monkeys, interhemispheric interactions have been described as primarily suppressive, in that simultaneous tactile stimulation of both hands suppresses neural activity in area 3b measured on one side through optical imaging Tommerdahl et al. (2006). Several studies have reported evidence for interhemispheric interactions in primary and secondary somatosensory cortex during bimanual stimulation (Hoechstetter et al., 2001; Krubitzer & Kaas, 1990; Reed et al., 2011).

In the model presented here, the first stage receives contra-lateral excitation and ipsi-lateral inhibition, assumed to be feed-forward tactile nerve fibre responses to stimulation on the right and left hand. The neural populations in the first stage of model make inter- and intra-hemispheric inhibitory connections with the neural populations in the second stage. As both stages of the model have bilateral inputs, area S2 is a good candidate for the neural populations and computations it describes (and/or higher areas downstream of S2). Neurons in area S2 have larger receptive fields and more complex response properties than their counterparts in area S1 Ruben et al. (2001). S2 is linked to higher level feature extraction and to cognitive functions such as attention and decision-making (Fitzgerald et al., 2006; Mima et al., 1998; Romo et al., 2002). Neurons within area S2 exhibit a tendency to encode both the stimulus and the behavioural outcome in a task-dependent way (Romo et al., 2002; Hernández et al., 2010). This task-dependent modulation and the correlation of neural activity with perceptual reports suggests a role of area S2 in sensory decision making. It is also possible that the first stage of model to be located at higher-order thalamus. Anterior pulvinar and mediodorsal thalamus project to S2 Disbrow et al. (2002), and higher-order thalamic areas have been linked to slowly changing state-like variables in tasks in rodents Wimmer et al. (2015).

### Stochastic influences on perceptual switching

In neural competition models, noise and adaptation processes are two possible switching mechanisms that account for perceptual alternations (Moreno-Bote et al., 2007; Chholak et al., 2020). In consideration of the experimental constraints on the statistics of alternations (mean of the dominance durations, their coefficient of variation and correlations between successive durations), models must operate with a balance between the noise and adaptation strength Shpiro et al. (2009). In several competition models, alternations are possible over large regions of parameter space, but the experimental constraints are satisfied in only a restricted domain where precise tuning of the system’s parameters is necessary Shpiro et al. (2009). A recent ensemble modeling of auditory streaming revealed smaller regions of parameter space are consistent with human data at the lower levels of auditory hierarchy Little et al. (2020).

The choice of stochastic process to reproduce the characteristics of perceptual rivalry including the short-tailed skewness of reversal time distributions has recently been under investigation Cao et al. (2014). It has also been shown that a generalized Ehrenfest stochastic process reproduces an experimentally-observed scaling property giving consistent ratios of distribution moments across a range of parameters, and the short-tailed skewness of reversal time distributions Cao et al. (2016). In the present study, we used an Ornstein-Uhlenbeck process, and the results produced by simulation of the tactile rivalry model were best fit by a log-normal distribution, consistent with a recent auditory and visual bistability study involving a large number of subjects Denham et al. (2018). The ratio of skewness and coefficient of variation for the distribution from the model are comparable to experimental values Darki and Rankin (2021). Looking at the distributions of perceptual durations from models provides insights for possible neural mechanism that drives these distributions. Models without adaptation, in which alternations are only driven by noise, produce exponential distributions. Adding adaptation can guarantee having a skewed shape log-normal distribution if the noise process fluctuates symmetrically, and a short-tailed gamma distribution if the noise process is asymmetric.

**Fig. 9:**
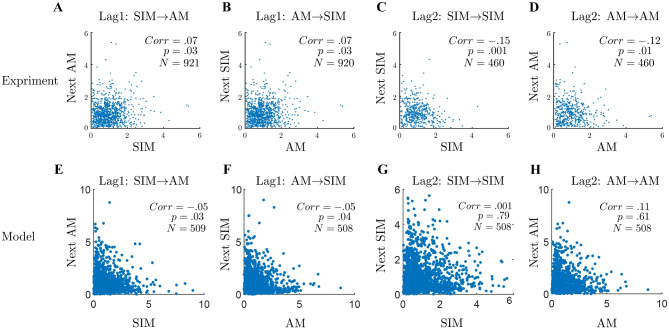
**A-D** Scatter plots of normalized durations from experiment. The correlation coefficient (*corr*) between perceptual phases for each scatter is indicated in each panel with the corresponding *p*-value and number of pairs (*p* and *N* respectively). **E-H** Scatter plots of normalized durations from model. The transition types are marked above each histogram (**A **& **E**) AM following SIM. (**B **& **F**) SIM following AM. (**C **& **G**) SIM. (**D **& **H**) AM

We found significant negative correlation for successive (lag 1) perceptual durations (from SIM to AM and vice versa) in the model. For perceptual durations that were one phase apart (lag 2), the correlation was not significantly different from zero (statistically independent, see Fig. 9 in Appendix). These results are in contrast with tactile rivalry experiments (significant positive correlation for lag 1 and negative correlation for lag 2) Darki and Rankin (2021). For the current model to capture statistical features of multistable perception including correlations, we would need to further explore the choice of noise processes. A recent hierarchical model of binocular rivalry uses out-of-equilibrium dynamics to reproduce dependence of durations on input strength, as well as the distribution of dominance durations and correlations Cao et al. (2021). A further investigation on the choice of stochastic process in the present tactile rivalry model could be done without the need to change other elements of model that work well (e.g. dependence of dominance durations on input strength).

### Predictions

Experimental data was used to constrain the model, in particular, the optimisation approach presented here determined the shape of the monotonically increasing, nonlinear relationship between $\Delta I$ and the first stage inputs (*D*). Equipped with this nonlinearity, the model can predict e.g. the dominance durations at values of $\Delta I$. It would be interesting to explore whether the model outperforms linear extrapolation between experimental data points. Furthermore, the nonlinearity predicted from the model may underpin other computations involving detection of differences in input intensity across the left and right fingers (or more generally at other locations). Indeed, the existence of a perceptual threshold for intensity discrimination with vibrotactile stimuli is known from behavioural experiments Gescheider et al. (1990). Low levels of intensity difference are not noticeable for participants, however, as we increase the intensity difference passing a threshold, participants are able to notice the intensity difference with performance levels saturating thereafter. Future experiments aimed at locating the first stage in the somatosensory pathway where such intensity differences are encoded would shed light on the encoding of two percepts considered in this study. Furthermore, our fitted model predicts the shape of the nonlinearity encoding intensity differences as a putative input to area S2.

In the model, the activity in the first stage is elevated (UP) when detected differences between left and right inputs (Fig. 2A) is transiently enhanced based on intrinsic noise in the population (Fig. 2B) and the current state of adaptation in the neural populations (not shown). The resulting effective enhancement of inhibition in the second stage leads to the SIM percept. Determining brain regions that encode differences between left and right tactile inputs would shed light on how the computations in the first stage are driven. This left-right difference and the activity that correlates with perception (as in Fig. 2C & D) and biases the encoded in the second stage (as in Fig. 2E) could feasibly be computed at secondary somatosensory cortex S2 (featuring bilateral receptive fields). Indeed, evidence from recordings in macaques viewing bistable stimuli show that the proportion of percept-related activity increases in higher (non-primary) visual areas Leopold and Logothetis (1999). As discussed above, lateral parietal cortex (area S2) could be involved in these computations, which could be investigated with non-invasive imaging. This is an experimentally testable prediction. For human participants, non-invasive imaging (EEG, MEG, fMRI) may not allow the spatial resolution to localise activity generated in a particular subdivision of somatosensory cortex. However, for the auditory system, the timing of activity associated with differences in perceptual interpretation can shed light on the putative origin of perceptual decisions Gutschalk et al. (2008). Moreover, recent work using large-scale intracranial recordings (in patients undergoing brain surgery) offers a unique opportunity to investigate this type of prediction Curtu et al. (2019).

### Future work, Levelt’s proposition IV

In this study, we only investigated Levelt’s proposition II, which considers the relation between dominant perceptual durations and asymmetric variation of feature difference (here $\Delta I$). Further experimental work needs to be done to demonstrate whether Levelt’s proposition IV also extends to tactile rivalry. This would provide an opportunity to further test and improve the model.

The model presented here has the flexibility to generate the first percept to be either SIM or AM, depending on the initial conditions. In order to tune the model to always start from SIM percept, we have chosen the initial states of the units in the first stage to be zero, which is a reasonable assumption. However, it is worth noting that our model does not produce the build-up characteristic of bistable perception, and further work is needed to account for this aspect of perceptual processing.

Further experimental work could investigate the effects of other features of the stimuli such as presentation rate (*PR*) and pulse durations (*TD*), drawing comparisons against the auditory streaming paradigm Pressnitzer and Hupé (2006). The application of bifurcation analysis to periodically forced rivalry models, originally presented in Darki and Rankin (2020) and utilised here, could be used to predict how the experimental result may be affected by the variation of these stimulus parameters.

The model presented here considers the computations of intensity difference at the first stage where contralateral excitations and ipsilateral inhibitions exist. Here the intensity difference between the high and low intensity pulses can also account for any attenuation of signals delivered from the other side of body. In auditory streaming when frequency and intensity differences of the tones delivered to the right and left ears are varied, both factors have a similar effect on neural activity in tonotopic responses, although the frequency difference has a larger effect than the intensity difference in auditory streaming Hartmann and Johnson (1991). It remains to be investigated whether a similar attenuation of signals from the opposite side of the body exists in somatosensation, and how stimuli from the right and left hands interact in detecting intensity differences between them.

The model works with the simple tactile rivalry stimuli delivered to the same locations on each hand. This model can be further developed to look at more complex stimuli such as tactile motion quartet involving four locations on the skin Carter et al. (2008).

## Conclusion

Earlier experimental work showed that perceptual rivalry extends to the tactile domain and common characteristics of multistable phenomena also persist with vibrotactile stimuli. This study presents a mathematical model for tactile rivalry based on physiological details from the somatosensory processing pathway. The proposed model is based on plausible neural mechanisms found throughout cortex and lower brain areas and captures the temporal and feature characteristics of perceptual interpretations for tactile rivalry. With parameter tuning model produces general characteristics of perceptual rivalry including Levelt’s proposition II, the short-tailed skewness of reversal time distributions and the ratio of distribution moments. The putative neural populations of the first stage and the second stage could be located within the secondary somatosensory cortex (area S2), or in higher cortical areas driven by activity in S2. The presented hierarchical model is generalisable and could be adapted to account for percept formation and competition leading to alternations for perceptually bistable stimuli from visual and auditory domains.

## Figures and Tables

**Fig. 7:**
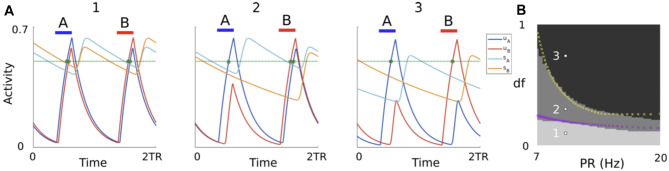
**A** Time histories of the 2TR-periodic states in the system defined in Eq. (14). Units’ threshold crossings are shown by green dots. **B** The total number of threshold crossings for both units is shown in greyscale for simulated trajectories at varying *PR* and *df* (black = 2, gray =3, lightest gray = 4 crossings). Parameters *PR* and *df* in panel (A) are shown by white dots in panel (B). The remaining parameters are $a = 2$, $b = 2.8$, $c = 5.5$, $\delta = 0.015$, $TD = 0.022$, $\tau _i = 0.25$, $\tau = 0.025$, $\theta = 0.5$. Adapted from Ferrario and Rankin (2021)

## Data Availability

Not applicable.

## Appendix

### Stage 1-encoding perceptual alternations

The dynamics of adapting recurrent model Levenstein et al. (2019) is described in terms of the mean firing rate $\nu$, and activity-driven adaptation $\alpha$ (Fig. 6A):

10 $$\begin{aligned} \begin{aligned}&{{\tau _\nu }\,{\dot{\nu }}} = - {\nu } + {N}\left( { w\,\nu - g\,\alpha + D} \right) ,\\&{\tau _\alpha }\,{{\dot{\alpha }}} = -\alpha + {A}\left( \nu \right) . \end{aligned} \end{aligned}$$

where *w* is the strength of recurrent excitation, *g* is the strength of adaptation, *D* is the level of input, and $\tau _\nu$ and $\tau _\alpha$ are time scales of mean firing rate and adaptation, respectively. *N*(*x*) and *A*(*x*) are assumed to be sigmoidal activation function as follows;

11 $$\begin{aligned} {N}(x) = \frac{1}{{1+e^{ -k_N(x - {x_0})}}} \end{aligned}$$

12 $$\begin{aligned} {A}(\nu ) = \frac{1}{{1+e^{ -k_A(\nu - {\nu _0})}}} \end{aligned}$$

Unless otherwise specified, we use ${x_0}=5$, ${\nu _0}=0.5$, $k_N=1$ and $k_A=15$ to parametrize the activation functions.

We used two of these adapting excitatory units in the fist stage of the full tactile rivalry model (Fig. 1D), one for the right and one for the left side (similar equations for variables $\nu _R$, $\alpha _R$ for the left side, and variables $\nu _L$, $\alpha _L$ for the left side). Where inputs $D_R$ and $D_L$ to these models are the stimulus differences from the right and left as follow;

13 $$\begin{aligned} \begin{aligned}&D_{R} = f(i_{L}- i_{R}),\\&D_{L} = f(i_{R}- i_{L}). \end{aligned} \end{aligned}$$

Function *f* is a nonlinearity, which will be determined later based on data-driven optimisation (see results section).

#### Stage 1-Bifurcation analysis

In the adapting recurrent model, UP/DOWN alternations are possible if there are coexisting stable states corresponding to UP and DOWN (i.e. bistability). This requires adequate strength of recurrent excitation *w*, to self-maintain the UP state under conditions of low drive. We show this first for a reduced case without adaptation dynamics ($g = 0$ in Eq. (10)).

The steady states of firing rate variable $\nu$, depend on the level of drive *D*, and the strength of recurrent excitation *w*. If recurrent excitation is weak ($w = 0$), the right-hand side of Eq. (10) increases monotonically with *D*, and has only one solution which is a stable fixed point (Fig. 6C). As recurrent excitation increases, the right-hand side of Eq. (10) shows a region of bistability between a low-rate bifurcation point at weak drive and a high-rate bifurcation point at strong drive (Fig. 6B). In the (*w*, *D*)-parameter plane, the bistable region (grey shaded area) has borders that correspond to saddle-node bifurcations (Fig. 6D). UP/DOWN bistability emerges at a critical value of recurrent excitation ($w = 4$), and a critical level of drive ($D = 3$). Consequently, a first general insight of adapting recurrent models is that UP/DOWN bistability will emerge in neuronal populations with sufficiently strong recurrent excitation, during conditions of low drive.

### Stage 2-encoding perceptual interpretation

The starting point for this model stage is a periodically driven competition network of two localised Wilson-Cowan units previously used for the encoding of different perceptual interpretations of the auditory streaming paradigm Ferrario and Rankin (2021). The model is described by the following system of delayed differential equations (DDEs):

14 $$\begin{aligned} \begin{aligned}&\tau {\dot{u}}_{R}=-{u_{R}(t)}+H(au_{L}(t)-bs_{L}(t-\delta )+ci_{R}(t)-\theta ),\\&{\dot{s}}_{R} =\dfrac{H(u_{R}(t)-\theta )(1-s_R(t))}{\tau }-\dfrac{s_{R}(t)}{\tau _i},\\&\tau {\dot{u}}_{L}=-{u_{L}(t)}+H(au_{R}(t)-bs_{R}(t-\delta )+ci_{L}(t)-\theta ),\\&{\dot{s}}_{L} =\dfrac{H(u_{L}(t)-\theta )(1-s_L(t))}{\tau }-\dfrac{s_{L}(t)}{\tau _i}. \end{aligned} \end{aligned}$$

where units $u_{R}$ and $u_{L}$ represent the mean firing rate of two neural populations encoding sequences of vibrotactile pulses with timescale $\tau$. The Heaviside gain function *H*(*x*) with activity threshold $\theta$ is given by

15 $$\begin{aligned} H(x) = \left\{ \begin{array}{l} {\begin{matrix} &{} 1 \,\,\,\, x>=0, \\ &{} 0 \,\,\,\, otherwise.\\ \end{matrix}} \end{array} \right. \end{aligned}$$

Mutual coupling through direct fast excitation has strength *a*. The delayed, slowly decaying inhibition has timescale $\tau _i$, strength *b* and delay $\delta$. The model is driven by excitatory inputs $i_R(t)$ and $i_L(t)$ with strength *c*. The synaptic variables $s_{R}$ and $s_{L}$ describe the time-evolution of the inhibitory dynamics.

#### Transformation to continuous model for bifurcation analysis

As shown in Ferrario and Rankin (2021), this system exhibits three different dynamical states relevant to perceptual interpretations of alternating stimuli (Fig. 7A). The states are distinguishable by the number of threshold crossings per 2*TR* period: (1) both units ($u_L$ and $u_R$) cross threshold in response to both input pulses (low and high) (total of 4 crossings, corresponds to integration in auditory streaming), (2) the *R* unit crosses threshold twice and the *L* unit once (total of 3 crossings, corresponds to bistability in auditory streaming), and (3) both units cross threshold once (total of 2 crossings, corresponds to segregation in auditory streaming).

The states (1) and (3) match the percepts observed in the experiments of tactile rivalry (SIM and AM, respectively). However, the state (2) does not have any specific meaning in the tactile domain (Fig. 7B). So, we are looking for a region in the parameter space that state (2) does not exist.

The model in Eq. (14) has been described using a system of DDEs. To be able to perform bifurcation analysis (using Auto07p), we need to transform it to a system of ODEs. For this aim, the dynamics of direct inhibitory synapses $s_R(t)$ and $s_L(t)$ is replaced with the dynamics of an indirect synapse (to describe the dynamics of $s_R(t-\delta )$ and $s_L(t-\delta )$). These indirect synapses are modelled by introducing two other synaptic variables $x_R(t)$ and $x_L(t)$ Rubin and Terman (2000) with dynamics described by:

16 $$\begin{aligned} \begin{aligned}&{\dot{s}}_{i} = \alpha _{s}{H(u_{i}-\theta _{u})(1-s_i)} - \beta _{s}{s_{i}} ,\\&{\dot{x}}_{i} = \alpha _{x}{H(s_{i}-\theta _s)(1-x_i)} - \beta _{x}{x_{i}}. \end{aligned} \end{aligned}$$

Here, $\alpha _{i}$ and $\beta _{i}$ ($i= s, x$) are positive constants. These indirect synapses have the effect of introducing a delay in the synaptic action, and this delay takes place on the slow timescale. Once $u_i$ crosses the threshold $\theta _{u}$, then $s_i$ will activate. The activation of $x_i$ must wait until $s_i$ crosses the second threshold $\theta _s$, thus introducing a delay.

We can choose $\alpha _{s}=\dfrac{1}{\tau }$, $\beta _{s}=\dfrac{1}{\tau _i}$, and $\theta _{u}=\theta$, and tune parameters $\alpha _{x}$, $\beta _{x}$, and $\theta _{s}$, so that the dynamics of $x_R(t)$ and $x_L(t)$ can generate delays approximately equal to the fixed delay $\delta$ in the system of DDEs (Eq. 14). Then $s_R(t-\delta )$ and $s_L(t-\delta )$ in Eq. (14) can be replaced with $x_R(t)$ and $x_L(t)$, respectively. Now the dynamics of the model can be described by the following system of ODEs:

17 $$\begin{aligned} \begin{aligned}&\tau \,{\dot{u}}_{R} = - {u_{R}} +H(a\,u_{L}-b\,x_{L}+c\,i_{R}-\theta ),\\&{\dot{s}}_{R} = \dfrac{H(u_{R}-\theta )(1-s_R)}{\tau } - \dfrac{s_{R}}{\tau _i} ,\\&{\dot{x}}_{R} = \alpha _x{H(s_{R}-\theta _{s})(1-x_R)} - \beta _x{x_{R}} ,\\&\tau \,{\dot{u}}_{L} = - {u_{L}} +H(a\,u_{R}-b\,x_{R}+c\,i_{L}-\theta ),\\&{\dot{s}}_{L} = \dfrac{H(u_{L}-\theta )(1-s_L)}{\tau } - \dfrac{s_{L}}{\tau _i},\\&{\dot{x}}_{L} = \alpha _x{H(s_{L}-\theta _{s})(1-x_L)} - \beta _x{x_{L}}. \end{aligned} \end{aligned}$$

#### Stage 2-Bifurcation analysis

Bifurcation analysis of the second stage with respect to intensity difference $\Delta I$ is shown in Fig. 8. The left and the right units receive inputs $i_L$ and $i_R$, which are antiphase sequences of high and low amplitude pulses ($\Delta I$ below the full amplitude pulse) with duration *TD* (Fig. 8A). There are reciprocal excitatory (with strength *a*) and inhibitory (with strength *b*) connections between the two units (Fig. 8B). Population activities $u_L$ and $u_R$ can encode the SIM percept, when $u_L$ and $u_R$ have a full response to both pulses (Fig. 8C). The AM percept is encoded when each unit only responds to the full amplitude input pulse and does not respond to the low amplitude input pulse (Fig. 8D). One parameter bifurcation diagrams are shown at three different values of mutual excitation *a*, with other parameters fixed ($b=2.8$, $c=5.5$). A blue curve shows the branch of periodic orbits, for which there is a sharp transition between periodic responses encoding the SIM and AM percepts (top panel in Fig. 8E). The boundary between these two dynamical behaviours moves to the right with higher values of excitation (middle panel), and to the left with lower values of excitation (bottom panel). When coupled to the first stage, the effective value of *a*, $a_{\text {eff}}=a-d\nu$ creates a hysteresis loop between these two cases (Fig. 3B).

### Full tactile rivalry model

To form the full tactile rivalry model, two units of adapting recurrent model encoding alternations at the first stage Eqs. (10–13) is incorporated with the model encoding percepts at the second stage Eq. (17). Units $\nu _R$ and $\nu _L$ in the first stage make inter- and intra-hemispheric inhibitory connections with strength *d* to the units $u_R$ and $u_L$ in the second stage (Fig. 1D).

18 $$\begin{aligned} \begin{aligned}&{\tau _\nu }{\dot{\nu }_R} = - {\nu _R} + N( w\nu _R- g\alpha _R + f(\Delta I)\frac{i_{L}- i_{R}}{\Delta I} +\zeta (t)),\\&{\tau _\alpha }\,{{\dot{\alpha }_R}} = -\alpha _R + {A}\left( \nu _R\right) ,\\&{\tau _\nu }{\dot{\nu }_L} = - {\nu _L} + N(w\nu _L- g\alpha _L + f(\Delta I)\frac{i_{R}- i_{L}}{\Delta I} +\zeta (t)),\\&{\tau _\alpha }\,{{\dot{\alpha }_L}} = -\alpha _L + {A}\left( \nu _L\right) ,\\&\tau {\dot{u}}_{R} = - {u_{R}} +H(au_{L}-bx_{L}+ci_{R}-d(\nu _R+\nu _L)-\theta ),\\&{\dot{s}}_{R} = \dfrac{H(u_{R}-\theta _{u})(1-s_R)}{\tau } - \dfrac{s_{R}}{\tau _i} ,\\&{\dot{x}}_{R} = \alpha _x{H(s_{R}-\theta _{s})(1-x_R)} -\beta _x{x_{R}} ,\\&\tau {\dot{u}}_{L} = - {u_{L}}+H(au_{R}-bx_{R}+ci_{L}-d(\nu _R+\nu _L)-\theta ),\\&{\dot{s}}_{L} = \dfrac{H(u_{L}-\theta _{u})(1-s_L)}{\tau } - \dfrac{s_{L}}{\tau _i},\\&{\dot{x}}_{L} = \alpha _x{H(s_{L}-\theta _{s})(1-x_L)} - \beta _x{x_{L}}. \end{aligned} \end{aligned}$$

### Simplified tactile rivalry model

As the inputs $(i_{L}- i_{R})$ and $(i_{R}- i_{L})$ to the full tactile rivalry model are antiphase and there is also symmetry in the first stage of the model, this model can be simplified, and units $\nu _R$ and $\nu _L$ can be replaced by one adapting recurrent model with variables $\nu$ and $\alpha$ and input $D=f(\Delta I)$ (Fig. 3A). Where $\Delta I$ is a positive constant, and *f* is a nonlinearity to be determined by data-driven optimisation. So the simplified tactile rivalry model is described by

19 $$\begin{aligned} \begin{aligned}&{\tau _\nu }\,{\dot{\nu }} = - {\nu } + N(w\,\nu - g\,\alpha + D +\zeta (t)),\\&{\tau _\alpha }\,{{\dot{\alpha }}} = -\alpha + {A}\left( \nu \right) ,\\&\tau \,{\dot{u}}_{R} = - {u_{R}} +H(a\,u_{L}-b\,x_{L}+c\,i_{R}-2\,d\,\nu -\theta ),\\&{\dot{s}}_{R} = \dfrac{H(u_{R}-\theta _{u})(1-s_R)}{\tau } - \dfrac{s_{R}}{\tau _i} ,\\&{\dot{x}}_{R} = \alpha _x{H(s_{R}-\theta _{s})(1-x_R)} - \beta _x{x_{R}} ,\\&\tau \,{\dot{u}}_{L} = - {u_{L}} +H(a\,u_{R}-b\,x_{R}+c\,i_{L}-2\,d\,\nu -\theta ),\\&{\dot{s}}_{L} = \dfrac{H(u_{L}-\theta _{u})(1-s_L)}{\tau } - \dfrac{s_{L}}{\tau _i},\\&{\dot{x}}_{L} = \alpha _x{H(s_{L}-\theta _{s})(1-x_L)} - \beta _x{x_{L}}. \end{aligned} \end{aligned}$$

## Code availability

Source code for the model is available in the GitHub repository farzaneh-darki/Darki2022-hierarchical: https://github.com/farzaneh-darki/Darki2022-hierarchical
